# Oncological and reproductive outcomes for gonadotropin-releasing hormone agonist combined with aromatase inhibitors or levonorgestrel-releasing intra-uterine system in women with endometrial cancer or atypical endometrial hyperplasia

**DOI:** 10.1136/ijgc-2022-003882

**Published:** 2022-11-11

**Authors:** Junyu Chen, Dongyan Cao, Jiaxin Yang, Mei Yu, Huimei Zhou, Ninghai Cheng, Jinhui Wang, Ying Zhang, Peng Peng, Keng Shen

**Affiliations:** 1 Department of Obstetrics and Gynecology, National Clinical Research Center for Obstetric and Gynecologic Diseases, Chinese Academy of Medical Sciences and Peking Union Medical College, Beijing, China; 2 Department of Obstetrics and Gynecology, Qilu Hospital of Shandong University, Jinan, Shandong, China; 3 Division of Gynecology oncology, Qilu Hospital of Shandong University, Jinan, China

**Keywords:** Carcinoma, Gynecology, Uterine Cancer, Endometrial Hyperplasia

## Abstract

**Objective:**

To evaluate the efficacy and safety of gonadotropin-releasing hormone agonist (GnRHa) combined with a levonorgestrel-releasing intrauterine device (LNG-IUD) or aromatase inhibitor (letrozole) in women with endometrial carcinoma or atypical endometrial hyperplasia who wished to preserve fertility.

**Methods:**

Patients at the Department of Obstetrics and Gynecology, Peking Union Medical College Hospital between January 2013 and December 2020 were retrospectively reviewed. A total of 179 patients who were unsuitable to undergo treatment with high-dose oral progestin, including those with progestin allergies, body mass index ≥30 kg/m^2^, liver and/or renal dysfunction, hypercoagulable state, and thrombosis were included. Patient data were retrieved from medical records and a prospectively maintained database that represented the standard protocol was followed for all patients. Clinical characteristics, treatment outcomes, adverse events, and reproductive outcomes were collected and analyzed. Logistic regression models were constructed to determine the associations between complete remission, recurrence, and fertility.

**Results:**

Overall, 169 patients (94.4%) achieved complete remission; 58 (96.7%) had atypical endometrial hyperplasia and 111 (93.3%) had endometrial carcinoma. The complete remission rates for the GnRHa plus LNG-IUD and GnRHa plus letrozole groups were 93.5% and 95.8%, respectively. The median time to complete remission was 6 (range 3–18) months: 4 (range 3–10) months for atypical endometrial hyperplasia and 8 (range 3–18) months for endometrial carcinoma. After a median follow-up of 27.5 (range 3–92) months, 41 (24.3%) women developed recurrence, with a median recurrence time of 17 (range 6–77) months. Of the patients with complete remission, 134 patients desired to conceive and 42 (32.3%) became pregnant, 24 (17.9%) were successfully delivered, 5 (3.7%) were still pregnant, while 13 miscarried.

**Conclusion:**

GnRHa combined treatment provides favorable oncological and reproductive outcomes. Larger multi-institutional studies are required to confirm these preliminary findings.

**WHAT IS ALREADY KNOWN ON THIS TOPIC**

- Fertility-sparing treatments such as oral progestins have been used in young patients with endometrial carcinoma and atypical endometrial hyperplasia. Adverse effects including weight gain, liver or renal dysfunction, and resistance to oral progestins may lead to treatment failure and alternative treatment options are required.

**WHAT THIS STUDY ADDS**

- The combination of GnRHa with LNG-IUD/aromatase inhibitors provides favorable oncological and reproductive outcomes, which are comparable to those of traditional oral progestin therapy.

**HOW THIS STUDY MIGHT AFFECT RESEARCH, PRACTICE, OR POLICY**

- GnRHa combined treatment is an effective fertility-sparing method for young women with endometrial carcinoma and atypical endometrial hyperplasia who are unsuitable for treatment with oral progestins, achieving a high rate of regression with minor side effects.

## Introduction

Endometrial cancer is one of the most common and progressively more problematic gynecological cancers, with a gradually increasing incidence in recent years.^1–3^ With the proportion of the obese population increasing, the incidence of endometrial cancer among relatively younger people is also increasing. Approximately 15% of endometrial cancers occur in pre-menopausal women, and 5% are of childbearing age.^4^ The standard treatment for this disease requires hysterectomy with bilateral salpingo-oophorectomy, with or without resection of the sentinel lymph nodes, pelvic or para-aortic lymphadenectomy. However, this standard treatment results in a permanent loss of fertility, but young patients have a strong desire to bear children. Therefore, conservative treatment should be considered in young patients with early-stage endometrial carcinoma or atypical endometrial hyperplasia who wish to preserve their fertility.

High-dose progestins including medroxyprogesterone acetate and megestrol acetate are the mainstay of conservative treatments for atypical endometrial hyperplasia and endometrial carcinoma.^5–7^ However, adverse effects such as weight gain, liver or renal dysfunction, and resistance to oral progestins may lead to treatment failure. Therefore, alternative treatment options are required. At present, gonadotropin-releasing hormone agonist (GnRHa) plus a levonorgestrel-releasing intra-uterine device (LNG-IUD) can be used as an alternative to oral/systemic progestin for the treatment of women with endometrial carcinoma and atypical endometrial hyperplasia.^8^ In addition, a combination of GnRHa and aromatase inhibitors has also been reported as an option for preserving fertility in women with endometrial carcinoma which achieved a favorable response.^9^ However, available evidence on the effective application of GnRHa-based combination treatment is limited. This study aimed to investigate the efficacy and safety of GnRHa plus LNG-IUD/aromatase inhibitors in young women with endometrial carcinoma or atypical endometrial hyperplasia.

## Methods

### Patients

All patients who received GnRHa-based therapy between January 2013 and December 2020 at the Department of Obstetrics and Gynecology, Peking Union Medical College Hospital were included. Patient information was collected from medical records and a prospectively maintained database which represented a standard protocol was followed for all patients. The inclusion criteria were as follows: (1) women aged 18–45 years who desired to preserve their fertility; (2) histologically confirmed atypical endometrial hyperplasia or early-stage endometrioid adenocarcinoma grade 1–2; (3) no signs of myometrial invasion or extra-uterine metastasis confirmed by enhanced magnetic resonance imaging (MRI); (4) unsuitable for high-dose oral progestin (fulfilling at least one of the following conditions: allergic to progestin; body mass index ≥30 kg/m^2^; liver and renal dysfunction; patients with hypercoagulable state, thrombophlebitis or thrombosis); (5) written informed consent provided by patient; and (6) regular follow-up, full text, and complete data available.

### Treatment Regimens

Two regimens were used: (1) GnRHa plus LNG-IUD: a combination of a subcutaneous injection of 3.75 mg GnRHa every 4 weeks and Mirena IUD (Bayer Healthcare Pharmaceutical) insertion; and (2) GnRHa plus aromatase inhibitors: a combination of a subcutaneous injection of 3.75 mg GnRHa every 4 weeks and oral letrozole 2.5 mg daily. Distribution of patients in these two groups was made based on the physicians’ recommendation and patients’ choices. GnRHa plus LNG-IUD has been recommended for patients with no birth plans in recent years. Women with a larger uterus, obesity and/or a history of polycystic ovary syndrome were recommended to receive GnRHa plus aromatase inhibitors.

During treatment, weight loss plans including diet control and exercise recommendations were provided to all patients, even for patients with a normal body mass index. Outpatient visits were arranged during treatment, symptoms such as vaginal spotting and abdominal pain were recorded, and physical examination including body weight and laboratory tests such as complete blood counts and biochemistry panels were performed. Transvaginal ultrasound was conducted at every visit to assess the endometrium. A histological response was determined by endometrial biopsy under hysteroscopic evaluation every 3–4 months (one course) during treatment.

### Response Evaluation

The pathological responses to treatment were categorized as complete response, partial response, stable disease, and progressive disease. Complete response was defined as the absence of evidence of hyperplasia or carcinoma; partial response was defined as regression of atypical endometrial hyperplasia or endometrial cancer to hyperplasia without atypia; stable disease was defined as the persistence of disease as initially diagnosed, or patients with signs of response to treatment, but with existence of the initial disease; and progressive disease was defined as progression to a lesion of higher grade or progressive disease including myometrial invasion, extra-uterine disease, or lymph node metastasis.^10^ Patients with a partial response or stable disease continued treatment for an additional 1–2 courses, whereas those with progressive disease were immediately recommended to undergo hysterectomy. Those who did not achieve complete remission after 12 months of therapy were considered to have failed fertility-preserving treatment and were recommended to undergo surgery. Once complete remission was achieved, patients who desired pregnancy were encouraged to conceive or were referred to undergo assisted reproductive technology. Patients in complete remission without birth plans were prescribed oral contraceptives, cyclic progestin, or LNG-IUD insertion to prevent recurrence.

### Follow-up

After complete remission, all patients were regularly followed up for a prolonged period of 3–6 month intervals. During each follow-up visit the following information was collected: menstruation period or abnormal vaginal bleeding, results of trans-vaginal ultrasound scan or MRI if necessary, and data relating to relapse (interval between complete remission and recurrence, diagnosis of recurrence, treatment, and survival outcomes). If the patient underwent hysterectomy, the reason for and the histological results of the surgery were also collected. Pregnancy was confirmed by a fetal heartbeat. Fertility outcomes, including time of gestation, use of assisted reproductive technology, and obstetric complications were also documented.

### Statistical Analyses

Statistical analyses were performed using IBM SPSS Statistics for Windows (version 22.0; IBM Corp, Armonk, New York, USA). Categorical variables are summarized in frequency tables, whereas continuous variables such as body mass index, time of complete remission, recurrence, and follow-up are presented as median (min–max). Frequency distributions were compared using χ^2^ or Fisher’s exact tests and median values were compared using the Mann–Whitney U test. Logistic regression models were constructed to determine the associations between factors and complete remission, recurrence, and fertility. For all statistical tests, differences were considered statistically significant at p<0.05.

## Results

### Demographics and Clinical Manifestations

The clinicopathological characteristics of the patients are summarized in Table 1. Sixty (33.5%) patients were diagnosed with atypical endometrial hyperplasia and 119 (66.5%) were diagnosed with endometrial carcinoma. The median age at diagnosis was 31 years (range 21–43). One hundred and sixty-three (91.1%) women were nulliparous, 48 (26.8%) had co-morbidities including polycystic ovary syndrome and/or endometriosis (one patinets combined with both co-morbidities), and 72 (40.2%) patients were obese (body mass index ≥30 kg/m^2^). Overall, 107 patients received the GnRHa+LNG IUD regimen and 72 patients received the GnRHa+aromatase inhibitors regimen. No significant difference was found between the baseline characteristics of the patients in the two treatment groups.

**Table 1:** Clinicopathologic characteristics of patients

Characteristics	Values, N (%)
N	179
Body mass index, kg/m^2^, median (range）	29.2 (17.7–46.1)
Obesity*	72 (40.2%)
Age, years, median (range）	31 (21–43)
Co-morbidity	
Polycystic ovary syndrome	40 (22.3%)
Endometriosis	9 (5.0%)
Histology	
Atypical endometrial hyperplasia	60 (33.5%)
Endometrial carcinoma	119 (66.5%)
Regimen	
GnRHa+LNG-IUD	107 (59.8%)
GnRHa+aromatase inhibitors	72 (40.2%)

*Body mass index ≥30 kg/m^2^.

GnRHa, gonadotropin-releasing hormone agonist; LNG-IUD, levonorgestrel-releasing intra-uterine device.

### Treatment Effects

In total, 169 (94.4%) patients achieved complete remission with a median complete remission time of 6 months (range 3–18) (Table 2). Two patients were diagnosed with grade 1–2 and all achieved complete remission. Ten (5.6%) patients who failed to achieve complete remission, including four with a partial response and six with stable disease, then underwent a hysterectomy. According to the post-operative pathological diagnosis, two were diagnosed with atypical endometrial hyperplasia, seven were diagnosed with stage IA endometrial cancer, and one with stage IC ovarian endometrial carcinoma. All patients were alive without tumors at the final contact after a median follow-up time of 27.5 months (range 3–92).

**Table 2:** Oncological outcomes of patients

Characteristics	Endometrial carcinoma (n=119)	Atypical endometrial hyperplasia (n=60)	Total (n=179)
Complete remission			
Complete remission rate	111 (93.3%)	58 (96.7%)	169 (94.4%)
Complete remission time, months, median (range)	8 (3–18)	4 (3–10)	6 (3–18)
Recurrence			
Recurrence rate	26 (23.4%)	15 (25.7%)	41 (24.3%)
Recurrence time, months, median (range)	16 (6–39)	28 (6–77)	17 (6–77)

The complete remission rate was 96.7% in patients with atypical endometrial hyperplasia and 93.3% in patients with endometrial carcinoma (p=0.351), and the median time to complete remission was 4 months (range 3–10) in patients with atypical endometrial hyperplasia and 8 months (range 3–18) in patients with endometrial carcinoma. At the end of the first course of treatment the complete remission rate of atypical endometrial hyperplasia was higher than that of endometrial carcinoma (65.0% vs 31.9%, p<0.0001). Similar results were observed in the second course (91.7% vs 72.3%, p=0.003) (Table 3). The complete remission rates in obese and non-obese patients were 95.3% and 93.1% (p=0.52), and 93.5% and 95.8% (p=0.34) in the GnRHa+LNG IUD and GnRHa+aromatase inhibitors groups, respectively. Patients with weight loss of >3% during the treatment period from the start of GnRHa injection to the end of the last evaluation had a higher response rate (98.8% vs 90.9%, p=0.023).

**Table 3:** Duration of complete remission

Times	Atypical endometrial hyperplasia	Endometrial carcinoma	Total	P value
1 course	65.0% (39)	31.9% (38)	43.0% (77)	**<0.0001**
2 courses	91.7% (55)	72.3% (86)	78.8% (141)	**0.003**
3 courses	96.7% (58)	89.1% (106)	91.6% (164)	0.145
Total	96.7% (58)	93.3% (111)	94.4% (169)	0.351

Values are shown as % (n).

### Adverse Effects

Post-menopausal symptoms such as hot flashes and vaginal dryness were the most common adverse effects (17.3%), followed by irregular bleeding (11.2%) and abnormal liver function (1.7%). The degree of menopausal symptoms was minor and no patients received add-back estrogen. IUD dislocation occurred in two patients and was resolved by reinsertion of the IUD. Scheduled treatment was not delayed because of these side effects. No treatment-related deaths occurred.

### Maintenance Therapy

After pathological complete remission was achieved, 147 patients received maintenance treatment including LNG-IUD, cyclical oral contraceptives, and low-dose cyclic progestin until they began attempting gestation. The remaining 22 patients did not receive maintenance therapy and were followed up regularly.

### Follow-up and Recurrence

After a median follow-up time of 27.5 months (range 3–92), 41 (24.3%) women developed recurrence (Table 2). The median time to recurrence was 17 months (range 6–77). Ten patients who gave up on preserving their uterus chose to undergo hysterectomy with or without lymphadenectomy. Extra-uterine lesions were identified in two patients, one with ovarian metastasis and one with para-aortic lymph node metastasis. The patient with lymph node metastasis was diagnosed with Lynch syndrome. These two patients received adjuvant chemotherapy and radiotherapy after endometrial cancer staging surgery. One was under follow-up after completing the recurrent therapy and the other remained in treatment.

Thirty-one patients received fertility-sparing retreatment after recurrence and 26 (83.9%) achieved complete remission. Hysterectomy was performed in three (9.7%) patients because of stable or progressive disease. The remaining three patients were still in treatment at the final contact. No patients died of the disease during this period. Factors related to recurrence are shown in Table 4. Multivariate analysis indicated that the recurrence rate was higher in patients over 30 years of age (32.3% vs 13.8%, p=0.011) and in those who lost <3% of their weight (32.2% vs 15.1%, p=0.019).

**Table 4:** Factors related to recurrence and pregnancy

Factors	Univariate analysis OR (95% CI)	P value	Multivariate analysis OR (95% CI)	P value
Recurrence				
Age (years): <30 vs ≥30	0.344 (0.159 to 0.746)	**0.005**	0.362 (0.165 to 0.794)	**0.011**
Atypical endometrial hyperplasia vs endometrial carcinoma	0.507 (0.247 to 1.024)	0.079		
Co-morbidity*: yes vs no	1.556 (0.728 to 3.324）	0.252		
Obesity: yes vs no	1.560 (0.871 to 2.794)	0.125		
Weight loss: <3% vs ≥3%	2.654 (1.234 to 5.658)	**0.010**	2.513 (1.162 to 5.434)	**0.019**
Maintenance therapy: no vs yes	1.481 (0.591 to 3.741)	0.400		
Pregnancy				
Age: <35 years vs >35 years	4.373 (1.427 to 13.406)	**0.010**	4.589 (1.472 to 14.307)	**0.009**
Co-morbidity: yes vs no	1.194 (0.513 to 2.782)	0.681		
Obesity: no vs yes	2.688 (1.284 to 6.203)	**0.018**	2.820 (1.215 to 6.545)	**0.012**
Atypical endometrial hyperplasia vs endometrial carcinoma	1.364 (0.604 to 3.079）	0.455		
Weight loss: ≥3% vs <3%	1.676 (0.800 to 3.512)	0.758		
In vitro fertilization and embryo transfer: yes vs no	2.231 (1.031 to 4.824)	**0.037**	2.098 (0.919 to 4.790)	0.055

*Combined with polycystic ovary syndrome and/or endometriosis.

### Fertility Outcomes

After achieving complete remission, 134 women attempted to conceive and 75 (56.0%) women were referred to receive assisted reproductive technology. In total, 42 (32.3%) patients became pregnant; 24 (17.9%) were successfully delivered, five (3.7%) were still pregnant, while 13 miscarried (nine were in the first trimester and four were in the second trimester). The median duration from complete remission to pregnancy was 12 months (range 1–72) (Table 5). In the univariate analysis the pregnancy rate was higher in patients aged <35 years (37.6% vs 12.1%, p=0.010). A higher probability was observed in non-obese patients than in obese patients (39.0% vs 19.23%, p=0.016). Assisted reproductive technology was associated with a high tendency for pregnancy (38.7% vs 13.7%, p=0.061), and a high pregnancy rate was observed in patients with in vitro fertilization and embryo transfer (47.8% vs 27.9%, p=0.037). Age and obesity remained significant in multivariate regression analysis (Table 4).

**Table 5:** Reproductive outcomes

Characteristics	Values, N (%)
Attempts to conceive	134
Natural conception	59 (44.0%)
Assisted reproductive technology	75 (56.0%)
Pregnancy	42 (32.3%)
Live baby delivery	24 (17.9%)
Ongoing	5 (3.7%)
Miscarriage	13 (9.7%)
Time from complete remission to pregnancy, months, median (range)	12 (1–72)

## Discussion

### Summary of Main Results

In this study we retrospectively analyzed 179 patients with endometrial carcinoma or atypical endometrial hyperplasia treated with GnRHa plus LNG-IUD/aromatase inhibitors. Overall, 94.4% of patients achieved complete remission with a 6-month median complete remission time. Approximately 24.3% of women developed recurrence and 32.3% became pregnant. The preliminary results are encouraging and comparable to those of previous oral progestin studies.^11 12^

### Results in the Context of Published Literature

Different conservative modalities have demonstrated safety and feasibility including oral progestins, LNG-IUD (alone or plus oral progestins), metformin (plus oral progestins), and hysteroscopic resection (plus oral progestins or LNG-IUD).^13^ Traditionally, the most frequently prescribed drugs for conservative endometrial cancer treatment are medroxyprogesterone acetate and megestrol acetate, and several studies have demonstrated their efficacy and safety.^13^ LNG-IUD represents a new delivery system for endometrial carcinoma treatment. It can provide local intra-uterine concentrations that are several-fold higher than those of oral progestins.^14^ Some researchers have used LNG-IUD alone or in combination with GnRHa and reported encouraging results.^9 15 16^

Metformin can promote progesterone receptor expression and has been used in combination with progestin for the conservative management of patients with endometrial cancer and atypical endometrial hyperplasia.^17^ The combination of hysteroscopic resection followed by progestin therapy has also been reported in fertility-sparing treatment, achieving a high response rate.^18^ GnRHa is an alternative to conservative treatment for endometrial diseases. Shan et al analyzed GnRHa+LNG-IUD in 57 patients compared with 40 patients treated with GnRHa alone. Patients receiving combination therapy exhibited the best disease-free survival.^19^ Letrozole is a third-generation aromatase inhibitor that can reduce estrogen levels by inhibiting estrogen synthesis, leading to a reduction in receptor-mediated growth stimulated in estrogen receptor-positive tumors such as endometrial carcinoma.^20 21^ The combination of GnRHa and letrozole has been reported to be an option for preserving fertility in women with endometrial carcinoma and atypical endometrial hyperplasia. Zhou et al administered GnRHa treatment combined with LNG-IUD or letrozole in 29 patients, 27 of whom achieved complete remission.^9^ Dong et al reported on three cases treated with GnRHa plus aromatase inhibitor in whom the disease remained stable for 2 years.^22^ In our previous study we used GnRHa combination therapy in 61 patients with obesity and 34 patients with recurrence, and achieved a high regression rate in these patients.^23 24^

In this study we have reported on a series of patients with endometrial carcinoma and atypical endometrial hyperplasia who were treated with GnRHa plus LNG-IUD/aromatase inhibitors. Over 90% of patients achieved complete remission (96.7% of patients with atypical endometrial hyperplasia and 93.3% of patients with endometrial carcinoma), indicating favorable effects. Most patients with atypical endometrial hyperplasia achieve complete remission within 6 months, but the median complete remission time for endometrial carcinoma is approximately 3–4 months longer. Eighteen patients achieved complete remission after an extension of the treatment time to 9–15 months. We therefore recommend that these combination regimens be administered for at least 6 months, especially for patients with endometrial carcinoma. However, the long-term adverse effects of GnRHa and its influence on fertility due to repeated curettage should be noted. Moreover, 2–3% of bone mass will be lost with 6 months’ use of GnRHa. Furthermore, there is uncertainty regarding the maximum duration of treatment, whether add-back treatment should be performed, whether the bone mineral density should be monitored, and whether calcium and bisphosphonates should be added.^25^ Considering that over 90% of patients with atypical endometrial hyperplasia achieved complete remission after two courses of treatments, and LNG-IUD could be used solely to treat endometrial carcinoma,^15 16^ for patients with atypical endometrial hyperplasia who already achieved partial response after 6 months the use of LNG-IUD alone might be an option to avoid the side effects of GnRHa. Nevertheless, its efficacy and safety are unclear and further research is required to accumulate more data.

In our study, patients who were obese and lost <3% body weight had lower response and pregnancy rates as well as higher recurrence rates, which was consistent with a previous study.^26^ Patients were unable to conceive due to obesity and polycystic ovary syndrome, which led to anovulation, and the absence of progestin stimulation may also increase the risk of recurrence.^27^ Weight control and health consultations that encourage increased exercise, better dietary habits, and changing lifestyles are crucial in the overall lifespan management of fertility-sparing treatment.

Previous studies have reported a high rate of relapse, ranging from 10% to 88%.^15^ In our study, approximately 25% of women experienced recurrence with a median recurrence time of 20 months. However, some recurrences were diagnosed as early as 6–7 months after complete remission. Another study reported that recurrence occurred 3–4 months after complete remission, which mandates the early start of follow-up.^28^ The latest recurrence in our cohort occurred at 7 years, while another study reported recurrence at 13 years.^29^ Therefore, long-term and regular follow-up is essential because of the high rate of late recurrence. In addition, we identified ovarian and lymph node metastases when recurrence occurred in our study. Patients should be informed of the risk of disease progression and occult lesions when choosing fertility-sparing treatment. In particular, fertility-sparing treatment may not be recommended for patients with Lynch syndrome because of the 100% recurrence rate reported.^30^ Additionally, hormonal maintenance therapy is important for complete responders who do not wish to conceive immediately after completing treatment.^31^ A low recurrence rate was also observed in pregnant patients. Therefore, immediate maintenance therapy and conception are encouraged to reduce the risk of recurrence.

The pregnancy and live birth rates in our study are lower than those reported in other large studies on progestin.^11 32^ This might be due to endometrium atrophy and decreased endometrial receptivity by repeated hysteroscopic evaluation and curettage. However, the miscarriage rate in the first or second trimester is similar to that in the general population.^33^ The follow-up time in our study was relatively short; if follow-up is performed for a longer time, a higher rate of relapse and live births may be observed.^34^ Despite the finding that assisted reproductive technology did not significantly improve the live birth rate, women who chose *in vitro* fertilization and embryo transfer had relatively better results, and some studies reported improved birth rates with assisted reproductive technology.^35^ Hence, once complete remission has been achieved, conception should be undertaken as soon as possible, and *in vitro* fertilization and embryo transfer is recommended without causing significant delays.

### Strengths and Weaknesses

To the best of our knowledge, the current study included the largest number of participants with both oncological and reproductive results regarding GnRHa-based treatments. These findings confirm that the combination of GnRHa with LNG-IUD/aromatase inhibitors is an effective method demonstrating a high rate of regression with minor side effects. However, there are still some limitations. First, this was a single-center retrospective study, and multi-center prospective clinical trials are required to verify the suitability of combination therapy using GnRHa with LNG-IUD/aromatase inhibitors for fertility preservation. Second, the follow-up time at this center was limited, and long-term follow-up of these patients should be performed to verify the high pregnancy rate reported. Third, although a previous study reported that the remission rate of LNG-IUD alone was lower than that in the present study,^14^ the lack of comparison between GnRHa combined therapy and LNG-IUD alone is still a limitation of this study, which is also the aim of our future work. Fourth, long-term side effects of GnRHa, such as osteoporosis and cardiovascular complications, should be considered in future studies.

### Implications for Practice and Future Research

Our research indicated that the combination of GnRHa with LNG-IUD/aromatase inhibitors is an effective fertility-sparing treatment with minor side effects, which could be an alternative method for patients who are unsuitable for high-dose oral progestin. Future larger-scale prospective studies or multi-center clinical trials are required to verify its efficacy. In addition, as The Cancer Genome Atlas-based molecular classification system is being introduced, it will be necessary to assess responses and recurrences in all future conservatively treated patients with endometrial carcinoma using this molecular perspective.

## Conclusion

The findings of our study confirm that the combination of GnRHa plus LNG-IUD/aromatase inhibitors is an effective method with a high rate of regression. The recurrence and pregnancy rates were comparable to those of traditional treatment with oral progestin. Therefore, the combination of GnRHa with LNG-IUD/aromatase inhibitors is a promising fertility-preserving alternative regimen for patients with endometrial carcinoma and atypical endometrial hyperplasia who are unsuitable for high-dose oral progestin, including those allergic to progestin or with body mass index ≥30 kg/m^2^, liver or renal dysfunction, hypercoagulable state, thrombophlebitis or thrombosis.

## Data Availability

Data are available upon reasonable request.

## References

[1] Lu KH , Broaddus RR . Endometrial cancer. N Engl J Med 2020;383:2053–64. 10.1056/NEJMra1514010 33207095

[2] Zhang S , Gong T-T , Liu F-H , et al . Global, regional, and national burden of endometrial cancer, 1990-2017: results from the Global Burden of Disease study, 2017. Front Oncol 2019;9:1440. 10.3389/fonc.2019.01440 31921687 PMC6930915

[3] Siegel RL , Miller KD , Jemal A . Cancer statistics, 2019. CA Cancer J Clin 2019;69:7–34. 10.3322/caac.21551 30620402

[4] Li M , Guo T , Cui R , et al . Weight control is vital for patients with early-stage endometrial cancer or complex atypical hyperplasia who have received progestin therapy to spare fertility: a systematic review and meta-analysis. Cancer Manag Res 2019;11:4005–21. 10.2147/CMAR.S194607 31190979 PMC6512613

[5] Gunderson CC , Fader AN , Carson KA , et al . Oncologic and reproductive outcomes with progestin therapy in women with endometrial hyperplasia and grade 1 adenocarcinoma: a systematic review. Gynecol Oncol 2012;125:477–82. 10.1016/j.ygyno.2012.01.003 22245711

[6] Guillon S , Popescu N , Phelippeau J , et al . A systematic review and meta-analysis of prognostic factors for remission in fertility-sparing management of endometrial atypical hyperplasia and adenocarcinoma. Int J Gynaecol Obstet 2019;146:277–88. 10.1002/ijgo.12882 31197826

[7] Yu M , Yang J-xin , Wu M , et al . Fertility-preserving treatment in young women with well-differentiated endometrial carcinoma and severe atypical hyperplasia of endometrium. Fertil Steril 2009;92:2122–4. 10.1016/j.fertnstert.2009.06.013 19591991

[8] Pashov AI , Tskhay VB , Ionouchene SV . The combined GnRH-agonist and intrauterine levonorgestrel-releasing system treatment of complicated atypical hyperplasia and endometrial cancer: a pilot study. Gynecol Endocrinol 2012;28:559–61. 10.3109/09513590.2011.649813 22296608

[9] Zhou H , Cao D , Yang J , et al . Gonadotropin-releasing hormone agonist combined with a levonorgestrel-releasing intrauterine system or letrozole for fertility-preserving treatment of endometrial carcinoma and complex atypical hyperplasia in young women. Int J Gynecol Cancer 2017;27:1178–82. 10.1097/IGC.0000000000001008 28562472

[10] Yu M , Wang Y , Yuan Z , et al . Fertility-sparing treatment in young patients with grade 2 presumed stage Ia endometrioid endometrial adenocarcinoma. Front Oncol 2020;10:1437. 10.3389/fonc.2020.01437 32983972 PMC7477323

[11] Chen M , Jin Y , Li Y , et al . Oncologic and reproductive outcomes after fertility-sparing management with oral progestin for women with complex endometrial hyperplasia and endometrial cancer. Int J Gynaecol Obstet 2016;132:34–8. 10.1016/j.ijgo.2015.06.046 26493012

[12] Yamagami W , Susumu N , Makabe T , et al . Is repeated high-dose medroxyprogesterone acetate (MPA) therapy permissible for patients with early stage endometrial cancer or atypical endometrial hyperplasia who desire preserving fertility? J Gynecol Oncol 2018;29:e21. 10.3802/jgo.2018.29.e21 29400014 PMC5823982

[13] Maggiore ULR , Khamisy-Farah R , Bragazzi NL , et al . Fertility-sparing treatment of patients with endometrial cancer: a review of the literature. J Clin Med 2021;10:4784. 10.3390/jcm10204784 34682906 PMC8539778

[14] Leone Roberti Maggiore U , Martinelli F , Dondi G , et al . Efficacy and fertility outcomes of levonorgestrel-releasing intra-uterine system treatment for patients with atypical complex hyperplasia or endometrial cancer: a retrospective study. J Gynecol Oncol 2019;30:e57. 10.3802/jgo.2019.30.e57 31074240 PMC6543108

[15] Pal N , Broaddus RR , Urbauer DL , et al . Treatment of low-risk endometrial cancer and complex atypical hyperplasia with the levonorgestrel-releasing intrauterine device. Obstet Gynecol 2018;131:109–16. 10.1097/AOG.0000000000002390 29215513 PMC5739955

[16] Minig L , Franchi D , Boveri S , et al . Progestin intrauterine device and GnRH analogue for uterus-sparing treatment of endometrial precancers and well-differentiated early endometrial carcinoma in young women. Ann Oncol 2011;22:643–9. 10.1093/annonc/mdq463 20876910

[17] Acosta-Torres S , Murdock T , Matsuno R , et al . The addition of metformin to progestin therapy in the fertility-sparing treatment of women with atypical hyperplasia/endometrial intraepithelial neoplasia or endometrial cancer: little impact on response and low live-birth rates. Gynecol Oncol 2020;157:348–56. 10.1016/j.ygyno.2020.02.008 32085863

[18] Yang B , Xu Y , Zhu Q , et al . Treatment efficiency of comprehensive hysteroscopic evaluation and lesion resection combined with progestin therapy in young women with endometrial atypical hyperplasia and endometrial cancer. Gynecol Oncol 2019;153:55–62. 10.1016/j.ygyno.2019.01.014 30674421

[19] Shan Y , Qin M , Yin J , et al . Effect and management of excess weight in the context of fertility-sparing treatments in patients with atypical endometrial hyperplasia and endometrial cancer: eight-year experience of 227 cases. Front Oncol 2021;11:749881. 10.3389/fonc.2021.749881 34804936 PMC8602817

[20] Barker LC , Brand IR , Crawford SM . Sustained effect of the aromatase inhibitors anastrozole and letrozole on endometrial thickness in patients with endometrial hyperplasia and endometrial carcinoma. Curr Med Res Opin 2009;25:1105–9. 10.1185/03007990902860549 19301987

[21] Azim A , Oktay K . Letrozole for ovulation induction and fertility preservation by embryo cryopreservation in young women with endometrial carcinoma. Fertil Steril 2007;88:657–64. 10.1016/j.fertnstert.2006.12.068 17428480

[22] Dong M , Jiang S , Tian W , et al . Preliminary clinical application of an aromatase inhibitor and a gonadotropin-releasing hormone agonist combination for inoperable endometrial cancer patients with comorbidities: case report and literature review. Cancer Biol Ther 2018;19:956–61. 10.1080/15384047.2018.1456609 29584567 PMC6301831

[23] Chen J , Cao D , Yang J , et al . Fertility-sparing treatment for endometrial cancer or atypical endometrial hyperplasia patients with obesity. Front Oncol 2022;12:812346. 10.3389/fonc.2022.812346 35251982 PMC8895268

[24] Chen J , Cao D , Yang J , et al . Management of recurrent endometrial cancer or atypical endometrial hyperplasia patients after primary fertility-sparing therapy. Front Oncol 2021;11:738370. 10.3389/fonc.2021.738370 34568074 PMC8458864

[25] Yang B-Y , Gulinazi Y , Du Y , et al . Metformin plus megestrol acetate compared with megestrol acetate alone as fertility-sparing treatment in patients with atypical endometrial hyperplasia and well-differentiated endometrial cancer: a randomised controlled trial. BJOG 2020;127:848–57. 10.1111/1471-0528.16108 31961463

[26] Shafiee MN , Chapman C , Barrett D , et al . Reviewing the molecular mechanisms which increase endometrial cancer (EC) risk in women with polycystic ovarian syndrome (PCOS): time for paradigm shift? Gynecol Oncol 2013;131:489–92. 10.1016/j.ygyno.2013.06.032 23822891

[27] Luo J , Chlebowski RT , Hendryx M , et al . Intentional weight loss and endometrial cancer risk. J Clin Oncol 2017;35:1189–93. 10.1200/JCO.2016.70.5822 28165909 PMC5455602

[28] Novikova OV , Nosov VB , Panov VA , et al . Live births and maintenance with levonorgestrel IUD improve disease-free survival after fertility-sparing treatment of atypical hyperplasia and early endometrial cancer. Gynecol Oncol 2021;161:152–9. 10.1016/j.ygyno.2021.01.001 33461741

[29] Wang C-J , Chao A , Yang L-Y , et al . Fertility-preserving treatment in young women with endometrial adenocarcinoma: a long-term cohort study. Int J Gynecol Cancer 2014;24:718–28. 10.1097/IGC.0000000000000098 24577149

[30] Britton H , Huang L , Lum A , et al . Molecular classification defines outcomes and opportunities in young women with endometrial carcinoma. Gynecol Oncol 2019;153:487–95. 10.1016/j.ygyno.2019.03.098 30922603

[31] Wang Y , Zhou R , Wang H , et al . Impact of treatment duration in fertility-preserving management of endometrial cancer or atypical endometrial hyperplasia. Int J Gynecol Cancer 2019;29:699–704. 10.1136/ijgc-2018-000081 30826750

[32] Chae SH , Shim S-H , Lee SJ , et al . Pregnancy and oncologic outcomes after fertility-sparing management for early stage endometrioid endometrial cancer. Int J Gynecol Cancer 2019;29:77–85. 10.1136/ijgc-2018-000036 30640687

[33] Ammon Avalos L , Galindo C , Li D-K ,. A systematic review to calculate background miscarriage rates using life table analysis. Birth Defects Res A Clin Mol Teratol 2012;94:417–23. 10.1002/bdra.23014 22511535

[34] Gallos ID , Gupta JK . Comment on: what about the relapse of endometrial hyperplasia? Eur J Obstet Gynecol Reprod Biol 2013;171:e5. 10.1016/j.ejogrb.2011.09.026 21992962

[35] Park J-Y , Seong SJ , Kim T-J , et al . Pregnancy outcomes after fertility-sparing management in young women with early endometrial cancer. Obstet Gynecol 2013;121:136–42. 10.1097/AOG.0b013e31827a0643 23262938

